# Use of Electronic Nicotine Delivery Systems among Adults with Mental Health Conditions, 2015

**DOI:** 10.3390/ijerph14010010

**Published:** 2016-12-23

**Authors:** Claire Adams Spears, Dina M. Jones, Scott R. Weaver, Terry F. Pechacek, Michael P. Eriksen

**Affiliations:** 1 Tobacco Center of Regulatory Science (TCORS), School of Public Health, Georgia State University, Atlanta, GA 30303, USA; djones153@student.gsu.edu (D.M.J.); srweaver@gsu.edu (S.R.W.); tpechacek@gsu.edu (T.F.P.); meriksen@gsu.edu (M.P.E.); 2 Division of Health Promotion and Behavior, School of Public Health, Georgia State University, Atlanta, GA 30303, USA; 3 Division of Epidemiology and Biostatistics, School of Public Health, Georgia State University, Atlanta, GA 30303, USA; 4 Division of Health Management and Policy, School of Public Health, Georgia State University, Atlanta, GA 30303, USA

**Keywords:** mental health conditions, electronic nicotine delivery systems, electronic cigarettes

## Abstract

Adults with mental health conditions (MHC) are especially likely to smoke and experience tobacco-related health disparities. Individuals with MHC may also use electronic nicotine delivery devices (ENDS) at disproportionately high rates. However, there is a relative dearth of knowledge regarding ENDS use among individuals with MHC. In a large representative sample of U.S. adults (*n* = 6051), associations between self-reported MHC diagnoses and ENDS use and susceptibility were examined, stratified by smoking status. Participants with MHC were approximately 1.5 times more likely to have used ENDS in their lifetime and almost twice as likely to currently use ENDS as those without MHC. MHC status was most strongly linked to higher ENDS use among former smokers, and former smokers with MHC were more likely to report using ENDS during past smoking quit attempts than those without MHC. Among participants who had not tried ENDS, former smokers with MHC were especially susceptible to future ENDS use. The potential advantage of ENDS for cessation purposes should be balanced with the risk of attracting former smokers with MHC to ENDS.

## 1. Introduction

Smoking is the leading cause of preventable mortality in U.S. and worldwide [1,2,3,4]. There is a critical need for innovative methods for harm reduction and smoking cessation, particularly among vulnerable populations such as smokers with mental health conditions (MHC) [5,6,7,8]. Over recent years, use of electronic nicotine delivery systems (ENDS; e.g., e-cigarettes) has increased dramatically in the general U.S. population, most notably among individuals who smoke combustible cigarettes (e.g., traditional cigarettes) [9,10]. ENDS could offer a lower risk alternative to traditional cigarettes and/or have utility as a cessation tool for current smokers [11,12,13,14,15,16]. Hence it is not surprising that smokers who use ENDS often report quitting or reducing cigarette smoking as primary motives [17,18,19]. However, some research has found that the use of e-cigarettes may be associated with a lower likelihood of quitting smoking [20]. More research is needed to rigorously evaluate the effectiveness of ENDS for smoking cessation [14,15,16] and, if they do have potential as a quitting aid, to understand any adverse effects including the possibilities of attracting youth or former smokers, renormalizing smoking, or maintaining addiction [21,22,23]. Unfortunately, there is a relative dearth of research on ENDS among individuals with MHC, a priority population with high overall smoking rates and at risk for profound tobacco-related health effects [5]. This study sought to document whether overall prevalence of ENDS use and rates of ENDS use in the context of smoking quit attempts differ for U.S. adults with or without MHC. Furthermore, potential risks of ENDS were examined by evaluating whether non-smokers and former smokers are considering using ENDS and whether individuals with MHC are more susceptible in this regard.

Although smoking rates have declined significantly in the general population [1,24], they remain disproportionately high among individuals with MHC [5,25,26], and associations between smoking and psychopathology have actually strengthened over time [27]. Smoking rates among adults with MHC are estimated to be more than double those of the U.S. general population and three to four times higher among those with conditions such as schizophrenia and bipolar disorder [5,7,26,28,29]. In a recent epidemiological survey, individuals who met criteria for at least one psychiatric disorder represented 36.4% of the U.S. adult population but smoked 56.4% of all cigarettes consumed [29]. Moreover, among smokers, those with MHC tend to smoke more heavily and have more difficulty quitting [7,28,30]. Individuals with MHC may smoke for self-medication; however the association between MHC and smoking is likely bidirectional and explained by a variety of underlying mechanisms [31].

Smokers with MHC are disproportionately burdened by the harm caused by cigarette smoking [5]. Approximately half of deaths among patients hospitalized with a primary psychiatric disorder are due to tobacco-related diseases [32]. Reviews of tobacco industry documents indicate that individuals with MHC have been specifically targeted, most notably by marketing to those with serious mental illness (SMI) and promoting smoking in psychiatric settings [33,34]. Smoking also imposes a significant financial burden on individuals with MHC, particularly those with SMI [5].

Fortunately, smokers with MHC often express interest in quitting [35], and smoking cessation is not only associated with reductions in depression and anxiety but also with increased quality of life among adults with MHC [36]. If deemed a safer and satisfying alternative to combustible cigarettes, ENDS could be a valuable tool for smokers with MHC [37]. Some emerging research suggests that ENDS have promise for harm reduction and/or smoking cessation, specifically among adults with MHC. In an uncontrolled pilot study, Caponnetto et al. [38] invited 14 smokers with schizophrenia who were not currently interested in quitting smoking to use e-cigarettes. After one year, nine (64.3%) of the participants had either reduced their daily cigarette consumption by half (seven participants) or quit smoking completely (two participants). In another pilot study, combustible tobacco use declined among 19 smokers with SMI who were provided e-cigarettes for four weeks [39]. In secondary analyses of a larger trial of ENDS for smoking cessation (*n* = 657, including 86 smokers with MHC), O’Brien et al. [40] reported that although smokers with MHC were more likely to relapse, the effects of e-cigarettes on quit rates did not differ by mental health status. Moreover, among smokers with MHC who did not quit, e-cigarettes containing nicotine were associated with greater reduction in cigarettes per day.

In the largest study of MHC and ENDS use to date, Cummins et al. [41] documented associations between ENDS use and MHC status (as classified by self-reported diagnosis of anxiety disorder, depression, or “other MHC”) using a 2012 national probability survey of U.S. adults (*n* = 10,041). Among current smokers, those with MHC were significantly more likely to be lifetime ENDS users than those without MHC (40% vs. 28.7%). Although not significant, there was a trend for current smokers with MHC to be more likely to currently use e-cigarettes than current smokers without MHC (8.6% vs. 5.4%). Among participants who had never tried e-cigarettes, current smokers with MHC indicated significantly greater susceptibility to e-cigarette use (i.e., higher self-reported likelihood of trying e-cigarettes in the future), compared to current smokers without MHC (60.5% vs. 45.3%). This 2012 study documented that adult smokers with MHC were more likely to use ENDS and were more susceptible to future ENDS use, compared to those without MHC. Continued surveillance of ENDS use over time in this priority population is warranted (e.g., the extent to which individuals with MHC are using ENDS specifically during attempts to quit smoking is unclear).

This study sought to build upon Cummins et al.’s [41] 2012 findings by examining 2015 data (given that patterns of ENDS use have changed dramatically over recent years [9]), by using more fine-grained MHC categories (including bipolar disorder and schizophrenia), by examining the extent to which smokers with MHC are using ENDS in smoking quit attempts (e.g., switching completely vs. partially to ENDS), and by more thoroughly assessing susceptibility to future ENDS use (e.g., likelihood of trying ENDS if offered by a friend). Our three primary aims were to examine whether adults with MHC exhibit different rates of: (1) use of ENDS (lifetime and current ENDS use); (2) use of ENDS specifically in the context of smoking quit attempts; and (3) susceptibility to future ENDS use. We hypothesized that individuals with MHC would be more likely to use ENDS and indicate higher likelihood of trying ENDS in the future compared to those without MHC. Based on Cummins et al.’s findings [41], it was expected that these associations would be strongest among current smokers.

## 2. Materials and Methods

### 2.1. Procedure and Sample

Data were collected through the 2015 Tobacco Products and Risk Perceptions Survey conducted by the Georgia State University Tobacco Center of Regulatory Science (TCORS). This is an annual, cross-sectional survey of a national random probability sample with an oversample of current smokers from GfK’s KnowledgePanel [42]. Survey participants were randomly selected with probabilities proportional to size (PPS) using a base panel weight after post-stratification adjustment for sampling and non-sampling sources of error (e.g., panel nonresponse and panel attrition).

GfK’s KnowledgePanel is a probability-based web panel designed to be representative of non-institutionalized U.S. adults. Adults sampled via address-based sampling or random digit dialing are eligible to join KnowledgePanel. Data collection occurred throughout August and September 2015. Participants completed the main survey in 25 min (median) and received a small cash-equivalent compensation for their participation. This study was approved by the Georgia State University Institutional Review Board (approval #H14028, 05/15/14).

Overall 8135 KnowledgePanel members were randomly selected and invited to participate in the survey: 7194 members for the general population sample, of whom 76.4% completed the screener survey and 5497 qualified for the main survey; and 941 members for the smoker oversample, of whom 72.6% completed the screener and 594 qualified for the main survey by confirming their current smoking status. Of the 6091 qualified completers, 38 cases from the general population sample and two cases from the smoker augment sample were excluded due to refusing to answer more than one-half of the survey questions, yielding an analytic sample of 6051 cases. A final stage completion rate of 74.0% and a qualification rate of 98.5% were obtained. A study-specific post-stratification weight was computed using an iterative proportional fitting (raking) procedure to adjust for survey non-response as well as for oversampling of smokers. Demographic and geographic distributions from the March 2015 Current Population Survey (CPS) were employed as benchmarks for adjustment, and included gender, age, race/ethnicity, education, household income, census region, metropolitan area, and Internet access.

### 2.2. Measures

#### 2.2.1. Demographic Characteristics

Respondent characteristics including sex, age, race/ethnicity, educational attainment, and annual household income were obtained from profile surveys administered by GfK to KnowledgePanel panelists.

#### 2.2.2. Mental Health Condition (MHC)

Self-reported MHC diagnoses were obtained from profile surveys administered by GfK to KnowledgePanel panelists. Panelists were asked if they had been diagnosed with a variety of medical conditions (“Have you been diagnosed by a doctor or other qualified medical professional with any of the following medical conditions?”), including the following MHCs: anxiety disorder, bipolar disorder, depression, mood disorder, schizoaffective disorder, schizophrenia, and other mental health conditions. Participants who indicated having been diagnosed with any of these conditions were coded as having a MHC (similar to the procedures of Cummins et al. [41], with the addition of bipolar disorder, mood disorder, schizoaffective disorder, and schizophrenia, which were not specifically assessed in that study). Participants were also asked whether they had ever seen a psychiatrist, psychologist, or social worker for counseling or therapy.

#### 2.2.3. Smoking Status

Respondents who reported smoking at least 100 cigarettes in their lifetime were asked, “Do you currently smoke cigarettes every day, some days, or not at all?’’. Those who responded “every day” or “some days” were considered current smokers, while those who responded “not at all” were considered former smokers. Those who reported that they had not smoked at least 100 cigarettes in their lifetime were considered non-smokers. These are commonly used indicators of current, former, and non-smoking status, (e.g., [43]).

#### 2.2.4. ENDS Use

Prior to receiving questions about ENDS use, participants were provided a description of electronic vapor products (e.g., e-cigarettes, e-cigars, e-hookahs, e-pipes, vape pens, hookah pens, or personal vaporizers/mods) and shown a picture of varied prototypical ENDS devices. Among participants who indicated that they were aware of ENDS, lifetime ENDS use was assessed by asking whether participants had ever used ENDS, even once or twice. Lifetime ENDS users were then asked, “Do you now use electronic vapor products every day, some days, rarely, or not at all?”. Those who responded “every day”, “some days”, or “rarely” were considered current ENDS users [44,45].

#### 2.2.5. ENDS Use during Smoking Quit Attempts

Current and former smokers were asked to report lifetime smoking cessation methods, including whether they had “switched completely” to ENDS or “substituted some” of their regular cigarettes with ENDS. These items were modified from the Evaluation of the National Tobacco Prevention and Control Public Education Campaign Smoker Questionnaire [46].

#### 2.2.6. Susceptibility to Future ENDS Use

Among participants who had not ever used ENDS, susceptibility to ENDS use was assessed with three items: (a) “Do you think you will try an electronic vapor product soon?”; (b) “Have you ever been curious about using an electronic vapor product?”; and (c) “If one of your best friends were to offer you an electronic vapor product, would you try it?”. Response options were “definitely yes”, “probably yes”, “probably not”, and “definitely not”. These items were adapted from Pierce’s susceptibility to smoking index [47], with the addition of an item assessing curiosity [48]. Items (a) and (c) have been used previously to assess susceptibility to e-cigarettes [49].

### 2.3. Statistical Analysis

Survey procedures within SAS 9.4 (SAS Institute Inc., Cary, NC, USA) were used to obtain weighted proportions and 95% confidence intervals for variables of interest, stratified by MHC and smoking status. Weighted logistic regression analyses were conducted predicting ENDS use from demographic characteristics (age, gender, race/ethnicity, income, and education), MHC, and their two-way interaction. There were not any significant interactions between MHC and demographic characteristics, and associations between MHC and ENDS use remained robust when demographic variables were in the model (consistent with the findings of Cummins et al. [41]). Therefore, the analyses reported here were from models excluding the interaction terms. Next, associations between MHC and outcomes, stratified by smoking status, were tested using weighted generalized linear models (logistic and linear regression) and Rao-Scott χ^2^ tests.

## 3. Results

### 3.1. Participant Characteristics and Associations between MHC and Smoking Status

Demographic characteristics of the total population and by MHC status are presented in Table 1. Participants who indicated having been diagnosed with MHC were more likely to be female (63.2% of participants with MHC were women, compared to 48.8% of those without MHC) and reported lower education and income than those without any MHC.

Overall, 18% indicated any lifetime MHC (see Table 2). The most common diagnosis was depression (13.4%), followed by anxiety disorders (8.6%). Among participants with MHC, 59.8% (95% CI: 56.0, 63.5) reported a single MHC, 26.2% (95% CI: 22.9, 29.6) reported two, and 14.0% (95% CI: 11.2, 16.8) reported three or more. Eighty percent (80.2% (95% CI: 77.2, 83.2)) of participants indicating MHC reported having sought mental health services (data not shown).

Table 2 also shows smoking rates by MHC. Among participants without MHC, most (60.2%) were non-smokers, and 12.2% were current smokers. Although the proportion of current smokers was higher among all MHCs, participants with schizophrenia and bipolar disorder were most likely to be current smokers. Most participants with schizophrenia (63.6%) and almost one-half of those with bipolar disorder (44.4%) were current smokers.

### 3.2. ENDS Use

Table 3 shows prevalence of ENDS use by MHC and smoking status. Seventeen percent (17.1% (95% CI: 15.9, 18.3)) of the total sample reported lifetime ENDS use, and current smokers (54.3% (95% CI: 50.3, 58.3)) were more likely than former (18.5% (95% CI: 16.1, 20.9)) and non-smokers (7.2% (95% CI: 6.0, 8.5)) to be lifetime ENDS users (data not shown). Participants with a MHC were more likely to have used ENDS than those without MHC (24.4% vs. 15.5%); Rao-Scott χ^2^ = 29.92, *p* < 0.001. The association between MHC status and lifetime ENDS use was most prominent among former smokers. Whereas approximately one-quarter (24.8%) of former smokers with a MHC reported lifetime use, 17.0% of those without a MHC had used ENDS, Rao-Scott χ^2^ = 5.45, *p* = 0.02. However, there were not significant associations between MHC and lifetime ENDS use specifically among current or non-smokers.

The number of MHC diagnoses reported was also positively related to lifetime ENDS use, Rao-Scott χ^2^ = 54.73, *p* < 0.001 (see Table 3). Specifically, 15.5% of participants with no MHC, 19.8% of those with one MHC, 25.3% of those with two MHCs, and 42.3% of those with three or more MHCs reported having used ENDS. The association between number of MHCs and lifetime ENDS use was most prominent among former smokers, Rao-Scott χ^2^ = 27.09, *p* < 0.001. In fact, almost two-thirds (64.4%) of former smokers with three or more MHCs reported having used ENDS.

Rates of current ENDS use in this sample were reported by Weaver et al. [50], who indicated that 7.4% (95% CI: 6.6, 8.4) of the total sample (29.8% (95% CI: 26.2, 33.5)) of current smokers, 5.3% (95% CI: 4.0, 7.1) of former smokers, and 3.0% (95% CI: 2.2, 3.9) of non-smokers were presently using ENDS. The current study found that participants with a MHC were more likely to report current ENDS use (11.4% of those with MHC vs. 6.6% without a MHC; Rao-Scott χ^2^ = 17.48, *p* < 0.001; see Table 3). As with lifetime ENDS use, the number of MHC diagnoses was associated with higher likelihood of current ENDS use, Rao-Scott χ^2^ = 36.67, *p* < 0.001. Specifically, 6.6% of people with no MHC, 9.3% of people with one MHC, 10.0% of people with two MHCs, and 22.8% of people with three or more MHCs reported currently used ENDS. Again, this association was strongest among former smokers, Rao–Scott χ^2^ = 12.61, *p* = 0.006.

Table 4 depicts prevalence of lifetime and current ENDS use by specific MHCs. All MHCs (except for schizophrenia) were related to higher likelihood of both lifetime and current ENDS use. ENDS use was most common among participants with schizoaffective disorder and bipolar disorder.

### 3.3. ENDS Use during Smoking Quit Attempts

Table 5 shows prevalence rates of ENDS use during past smoking quit attempts among current and former smokers, by mental health status. Overall, participants with MHC were more likely to have switched completely to ENDS in a past smoking quit attempt, Rao-Scott χ^2^ = 8.04, *p* = 0.005. This was most prominent among former smokers, Rao-Scott χ^2^ = 9.96, *p* = 0.002. Former smokers with MHC were over twice as likely to report switching completely to ENDS in a past smoking quit attempt (11.4% of former smokers with MHC vs. 5.3% of those without MHC).

Participants with MHC were also more likely to indicate having substituted some regular cigarettes with ENDS, Rao-Scott χ^2^ = 10.05, *p* = 0.002 (see Table 5). Again, this was most apparent among former smokers, Rao-Scott χ^2^ = 6.56, *p* = 0.01. Former smokers with MHC were almost twice as likely to report substituting some cigarettes with ENDS in a past smoking quit attempt (13.7% of former smokers with MHC vs. 7.3% of those without MHC). There were not significant associations between MHC and having ever switched completely or partially to ENDS among current smokers.

### 3.4. Susceptibility to Future ENDS Use

Table 6 shows susceptibility to future ENDS use among participants who had never tried ENDS. Overall, participants with MHC were more likely to say that they would “definitely” or “probably” try ENDS soon, compared to those without MHC, Rao-Scott χ^2^ = 7.19, *p* = 0.007. Although there was not a significant association between MHC and trying ENDS soon among non-smokers, MHC status was associated with higher interest in trying ENDS soon among both current (Rao-Scott χ^2^ = 4.37, *p* = 0.037) and former smokers (Rao-Scott χ^2^ = 7.73, *p* = 0.005). Whereas 26.9% of current smokers and 1.8% of former smokers with MHC reported that they thought they would try ENDS soon, 13.8% of current smokers and 0.3% of former smokers without MHC indicated the same likelihood of trying ENDS soon.

Participants with MHC were also more likely to say that they have been curious about using ENDS, Rao-Scott χ^2^ = 20.03, *p* < 0.001. This association was significant among current and former (but not non-) smokers. About half (49.4%) of current smokers with MHC indicated that they have been “definitely” or “probably” curious about trying ENDS, compared to 33.4% of those without MHC, Rao-Scott χ^2^ = 4.29, *p* = 0.038. Whereas 13.7% of former smokers with MHC indicated high curiosity about ENDS, 5.9% of former smokers without MHC indicated this same level of curiosity, Rao-Scott χ^2^ = 10.13, *p* = 0.002.

Participants with MHC also indicated greater likelihood of trying ENDS if offered by one of their best friends, Rao-Scott χ^2^ = 20.6, *p* < 0.001. However, this was only significant among former smokers. Former smokers with MHC were almost three times more likely to say that if offered them by a best friend, they would “definitely” or “probably” try ENDS (9.4% vs. 3.3%, Rao-Scott χ^2^ = 14.47, *p* = 0.0001), as compared to their counterparts without MHC.

## 4. Discussion

Given the striking disparities experienced by smokers with MHC [5,6] and the relative dearth of knowledge regarding ENDS use among individuals with these conditions, this study documented ENDS usage and susceptibility among adults with vs. without MHC. As expected, in this large representative sample of U.S. adults, participants with MHC were more likely to report lifetime and current ENDS use. Based on previous findings [41], it was hypothesized that this association would be most pronounced among current smokers. However, MHC status was most strongly linked to greater likelihood of lifetime ENDS use among former smokers, who indicated higher rates of using ENDS in a past smoking quit attempt. Additionally, among participants who had not tried ENDS, those with MHC indicated greater likelihood of trying ENDS in the future (especially among former smokers).

This study documented alarmingly high smoking rates among individuals with MHC, highlighting the urgent need to identify and disseminate effective cessation interventions for these populations [5,6,7,8]. Although ENDS may offer potential as a tool for harm reduction and/or smoking cessation, concern has been raised about whether ENDS might widen existing disparities in smoking. For example, the diffusion of new, health-relevant technological innovations is often slower for those who are poorer and less educated, in part due to the often higher costs of new technologies [51]. Consistent with this concern, recent research has found that smokers with higher education are more likely to also use ENDS and that among dual users, those with a college education reported higher quit intentions and quit attempts than those with high school or less education [52]. Another study found that when adjusting for education, those with incomes above $50,000 were more likely to use ENDS [53]. Considering that those with mental illness are more likely to experience socioeconomic inequalities [54], these concerns and questions about the harm reduction potential of ENDS and their impact on tobacco-related disparities naturally extend to populations with mental illness. The findings of the present study provide some insight into these concerns and questions. 

Overall, participants with MHC were approximately 1.5 times more likely to have used ENDS in their lifetime and almost twice as likely to currently use ENDS as those without MHC. Given that former smokers with MHC were especially likely to have used ENDS during past smoking quit attempts, ENDS could provide a promising tool to reduce tobacco-related disparities for this population. If ENDS are an effective quitting aid, smokers with MHC may have much to gain. However, more research will be needed to clarify whether ENDS use reliably promotes smoking cessation for adults with specific MHCs. Small studies on ENDS use among smokers with MHC show promise, but results of larger studies in the general population have been mixed, and more rigorous research is needed [11,14,15,16,20]. Furthermore, it will be important to ascertain whether ENDS are viewed as a more or less satisfying alternative to combustible cigarettes among individuals with MHC.

MHCs are often comorbid with one another, and comorbidity (vs. having a single diagnosis) has been associated with greater severity and disability (e.g., [55,56,57]). In the current study, the number of self-reported MHC diagnoses was associated with higher likelihood of ENDS use. In fact, among former smokers, those reporting three or more MHCs were almost four times more likely to have used ENDS compared to those without any MHC. There are several possible reasons why individuals with more psychiatric symptoms or greater impairment might be most likely to use ENDS. For example, given that addictive disorders and other MHCs likely involve shared neurobiological pathways [58,59,60], having a greater number of comorbid disorders might reflect a stronger genetic vulnerability to addiction. In addition, former smokers with more MHCs might be especially likely to use ENDS as a way to self-medicate psychiatric symptoms or offset medication side effects once they are no longer receiving nicotine through combustible cigarettes. However, relationships between MHCs and nicotine are complex, and comorbid disorders may have not only additive but also interactive effects [56,61]. More research is needed to understand how specific psychiatric symptoms, symptom severity, and level of disability are related to ENDS use.

Although we cannot make direct comparisons between our results and those presented by Cummins et al. [41] because of some methodological differences (e.g., the current study assessed additional MHC categories; Cummins et al. examined sub-categories of former smokers), it is worth noting that the overall differences in ENDS use for U.S. adults with vs. without MHC were not as pronounced in our data. In Cummins et al.’s 2012 data [41], lifetime ENDS use rates for adults with vs. without MHC were 14.8% vs. 6.6% (adults with MHC were more than twice as likely to be lifetime ENDS users than those without MHC), and current ENDS use rates were 3.1% vs. 1.1% (those with MHC were almost three times as likely to currently use ENDS). In our 2015 data, rates of ENDS use for participants with vs. without MHC were 24.4% vs. 15.5% (lifetime use) and 11.4% vs. 6.6% (current use). Although longitudinal studies will be necessary to statistically compare these trends over time, it is possible that the gap between adults with vs. without MHC has narrowed slightly. It could be that individuals with MHC were more likely to be early adopters of ENDS when they first became available, but that the gap has narrowed as ENDS use has become more common across the general population.

Although ENDS might have utility for helping current smokers to quit, their potential to attract non-smokers and former smokers has been raised as a concern [21]. Our results suggest that former smokers with MHC might be a particularly vulnerable population in this regard. Among participants who had not tried ENDS, former smokers with MHC were six times more likely to indicate high likelihood of trying ENDS soon, over twice as likely to be highly curious about trying ENDS, and almost three times more likely to indicate high likelihood of trying ENDS if offered by a friend, compared to former smokers without MHC. The finding that former smokers with MHC are more susceptible to initiating ENDS use (Table 6) is of concern. The potential advantage of ENDS for reducing the high smoking rates among those with MHC should be balanced against the risk of attracting former smokers with MHC.

The findings must be interpreted in the light of several limitations. This study relied on self-reports of MHC, ENDS use, and smoking status, without clinical diagnoses or biochemical confirmation of smoking. Furthermore, participants reported whether or not they had ever been diagnosed with a MHC. Thus, individuals classified as having a MHC may or may not currently have the condition. However, the majority of participants who indicated MHC diagnoses did report that they had sought mental health services, thus increasing our confidence that self-reported MHCs were valid and clinically significant. In addition, analyses of specific MHCs, particularly schizophrenia, may be less reliable due to smaller sample sizes. However, this study provides the latest available national estimates on an understudied research area. Future research should consider using clinical interviews to determine specific psychiatric diagnoses (and their time frames) using the Diagnostic and Statistical Manual of Mental Disorders (DSM-5; [62]). Although the rates of several disorders in the current study are comparable to estimates from extant research (e.g., [41,62]), the National Comorbidity Survey Replication (NCS-R) found a higher prevalence rate for lifetime psychiatric disorders (46.4%, compared to 18.0% in the current study) [63] (NCS-R used diagnostic interviews and likely identified people with MHCs who had not been formally diagnosed by a medical provider). Future studies might also assess conditions not included in the current dataset, including substance use disorders or personality disorders.

This study is subject to the inherent limitations of cross-sectional data. Longitudinal research will be critical for understanding prospective associations between ENDS use and cessation among people with vs. without MHC, and randomized controlled trials of ENDS for smoking cessation (vs. more traditional treatment methods like nicotine replacement therapy and counseling) will enhance our understanding of whether ENDS are differentially effective as a cessation tool for individuals with vs. without specific MHCs. Finally, because this study examined a novel topic and it was deemed important to delve into associations between MHC and ENDS use separately by smoking status, a relatively large number of analyses were conducted. Given the potential for this approach to inflate Type I error, we suggest that readers examine point estimates and confidence intervals in addition to *p*-values. Replication will be needed to increase confidence in the findings.

## 5. Conclusions

Despite the above limitations, this study is one of the first to document ENDS use among adults with MHC, an understudied and high-priority population. Results from this large, nationally representative study suggest that adults with MHC, particularly former smokers, are more likely to use ENDS, and former smokers with MHC are more likely to report having used ENDS during smoking quit attempts than those without MHC. Among participants who had not tried ENDS, former smokers with MHC indicated a higher likelihood of trying ENDS in the future compared to those without MHC. Ongoing research should examine the efficacy of ENDS as a quitting aid among smokers with MHC, while also evaluating whether former smokers and non-smokers with MHC are being attracted to start using ENDS. 

## Figures and Tables

**Table 1:** Demographics characteristics (%) by lifetime mental health condition.

Demographic Characteristic	Overall (*n* = 6016)	Mental Health Condition (*n* = 1082)	No Mental Health Condition (*n* = 4934)
Gender ***			
Female	51.4 (49.8, 53.0)	63.2 (59.5, 66.8)	48.8 (47.0, 50.6)
Age			
18–29	21.2 (19.7, 22.7)	21.8 (18.6, 25.0)	21.1 (19.4, 22.7)
30–44	25.1 (23.7, 26.5)	26.7 (23.4, 30.0)	24.7 (23.1, 26.2)
45–59	27.0 (25.5, 28.4)	28.6 (25.1, 32.0)	26.2 (25.0, 28.2)
60+	26.8 (25.4, 28.1)	22.9 (19.9, 26.0)	27.6 (26.1, 29.1)
Race/Ethnicity **			
White, NH	66.1 (64.5, 67.7)	69.2 (65.4, 72.9)	65.4 (63.6, 67.3)
Black, NH	11.3 (10.1, 12.4)	8.6 (6.1, 11.1)	11.9 (10.6, 13.1)
Other, NH	7.4 (6.4, 8.4)	5.0 (3.2, 6.7)	7.9 (6.7, 9.1)
Hispanic	15.2 (14.0, 16.5)	17.3 (14.2, 20.4)	14.8 (13.4, 16.1)
Education ***			
<High School	11.2 (9.9, 12.5)	16.4 (12.8, 20.0)	10.1 (8.7, 11.4)
High School	30.1 (28.6, 31.5)	32.6 (29.2, 36.0)	29.5 (27.9, 31.1)
Some College	28.9 (27.4, 30.4)	27.7 (24.3, 31.0)	29.2 (27.5, 30.9)
≥Bachelor’s Degree	29.8 (28.4, 31.2)	23.4 (20.5, 26.3)	31.2 (29.6, 32.8)
Income ***			
<$15,000	11.7 (10.5, 12.8)	22.8 (19.3, 26.3)	9.2 (8.1, 10.4)
$15,000–$24,999	6.2 (5.4, 6.9)	8.6 (6.7, 10.6)	5.6 (4.8, 6.4)
$25,000–$39,999	14.9 (13.7, 16.1)	16.0 (13.1, 18.9)	14.7 (13.4, 15.9)
$40,000–$59,999	15.8 (14.6, 16.9)	15.9 (13.2, 18.6)	15.7 (14.5, 17.0)
$60,000–$84,999	19.0 (17.8, 20.4)	14.5 (12.0, 17.0)	20.1 (18.7, 21.5)
$85,000–$99,999	9.0 (8.0, 10.0)	6.5 (4.6, 8.3)	9.6 (8.4, 10.7)
$100,000+	23.4 (22.1, 24.8)	15.7 (13.1, 18.2)	25.1 (23.6, 26.7)

Weighted percentages are reported, with 95% confidence intervals in parentheses. Asterisks indicate significant associations as determined by Rao-Scott χ^2^ tests, ** *p* ≤ 0.01; *** *p* ≤ 0.001.

**Table 2:** Smoking status percentages by lifetime mental health condition.

Mental Health Condition (MHC)	*n*	% of Total Sample with Each MHC	Smoking Status		
			% Non-Smokers (*n* = 3060)	% Former Smokers (*n* = 1687)	% Current Smokers (*n* = 1282)
Any Mental Health Condition ***	1170	18.0 (16.7, 19.2)	47.3 (43.5, 51.1)	29.5 (26.0, 32.9)	23.2 (20.1, 26.3)
Bipolar Disorder ***	147	2.4 (1.8, 2.9)	33.0 (21.8, 44.3)	22.6 (11.7, 33.4)	44.4 (32.9, 55.9)
Schizoaffective Disorder	19	0.4 (0.1, 0.6)	48.8 (13.8, 83.7)	17.2 (0.0, 40.4)	34.0 (3.7, 64.4)
Schizophrenia ***	22	0.4 (0.2, 0.6)	7.6 (0.0, 16.9)	28.8 (4.8, 52.8)	63.6 (38.8, 88.5)
Anxiety Disorder ***	567	8.6 (7.7, 9.6)	45.5 (40.0, 51.1)	28.0 (23.0, 33.1)	26.4 (21.7, 31.1)
Depression ***	874	13.4 (12.3, 14.5)	46.4 (42.0, 50.8)	30.2 (26.2, 34.3)	23.4 (19.8, 27.0)
Mood Disorder ***	138	2.3 (1.7, 2.8)	44.4 (32.4, 56.3)	21.4 (11.6, 31.2)	34.2 (23.2, 45.2)
Other Mental Health Condition ***	121	1.6 (1.2, 1.9)	38.6 (27.7, 49.4)	29.8 (18.5, 41.1)	31.6 (20.9, 42.4)
No Mental Health Condition	4859	82.0 (80.8, 83.3)	60.2 (58.4, 61.9)	27.6 (26.1, 29.2)	12.2 (11.1, 13.3)

Unweighted frequencies (*n*) and weighted prevalence rates are reported. 95% confidence intervals are provided in parentheses. Asterisks indicate significant Rao-Scott χ^2^ tests comparing smoking status among participants reporting each MHC versus participants who did not report any MHC, *** *p* ≤ 0.001.

**Table 3:** Lifetime and current use of Electronic Nicotine Delivery Systems (ENDS) by lifetime mental health condition and smoking status.

Mental Health Condition	Overall	Non-Smokers (*n* = 3025)	Former Smokers (*n* = 1686)	Current Smokers (*n* = 1278)
Lifetime ENDS use	***		*	
Any Mental Health Condition	24.4 (21.1, 27.7)	7.7 (4.9, 10.5)	24.8 (18.0, 31.5)	57.1 (49.6, 64.6)
No Mental Health Condition	15.5 (14.2, 16.8)	7.1 (5.8, 8.5)	17.0 (14.5, 19.5)	53.1 (48.4, 57.9)
# of Mental Health Conditions	***		***	
0 Mental Health Conditions	15.5 (14.2, 16.8)	7.1 (5.8, 8.5)	17.0 (14.5, 19.5)	53.1 (48.4, 57.9)
1 Mental Health Condition	19.8 (16.1, 23.5)	7.1 (3.5, 10.7)	19.5 (12.4, 26.6)	55.1 (44.8, 65.4)
2 Mental Health Conditions	25.3 (18.8, 31.7)	8.9 (3.5, 14.2)	22.7 (11.4, 33.9)	60.6 (45.9, 75.2)
3+ Mental Health Conditions	42.3 (31.2, 53.4)	8.9 (0.5, 17.3)	64.4 (41.4, 87.3)	57.3 (41.7, 72.9)
Current ENDS use	***			
Any Mental Health Condition	11.4 (9.0, 13.8)	3.5 (1.4, 5.6)	6.7 (2.6, 10.7)	33.2 (26.1, 40.3)
No Mental Health Condition	6.6 (5.7, 7.5)	2.9 (2.0, 3.8)	5.1 (3.5, 6.8)	28.4 (24.2, 32.6)
# of Mental Health Conditions	***		**	
0 Mental Health Conditions	6.6 (5.7, 7.5)	2.9 (2.0, 3.8)	5.1 (3.5, 6.7)	28.4 (24.2, 32.6)
1 Mental Health Condition	9.3 (6.6, 12.0)	4.0 (1.1, 6.9)	4.6 (1.5, 7.7)	31.7 (22.1, 41.2)
2 Mental Health Conditions	10.0 (5.6, 14.4)	3.2 (0.3, 6.2)	4.7 (0.1, 9.4)	30.9 (16.2, 45.5)
3+ Mental Health Conditions	22.8 (13.1, 32.5)	1.0 (0.0, 2.9)	25.5 (0.0, 55.1)	38.1 (23.4, 52.8)

Weighted prevalence rates are reported, with 95% confidence intervals in parentheses. # = Number. Asterisks indicate significant associations between MHC and ENDS use among the overall sample and by smoking status as determined by Rao-Scott χ^2^ tests, * *p* < 0.05; ** *p* ≤ 0.01; *** *p* ≤ 0.001.

**Table 4:** Prevalence of lifetime and current use of Electronic Nicotine Delivery Systems (ENDS) by specific lifetime mental health conditions.

Mental Health Condition	*n*	Lifetime ENDS Use	Current ENDS Use
Any Mental Health Condition	1162	24.4 (21.1, 27.7) ***	11.4 (9.0, 13.8) ***
Bipolar Disorder	145	40.0 (28.4, 51.7) ***	21.3 (11.2, 31.3) ***
Schizoaffective Disorder	17	49.6 (16.7, 82.4) **	28.6 (0.0, 63.4) *
Schizophrenia	22	25.4 (2.0, 48.8)	7.4 (0.0, 20.4)
Anxiety Disorder	564	29.8 (24.6, 35.0) ***	13.4 (9.6, 17.2) ***
Depression	870	23.7 (20.0, 27.5) ***	11.0 (8.2, 13.8) **
Mood Disorder	136	29.6 (18.7, 40.5) **	13.9 (6.1, 21.7) *
Other Mental Health Condition	119	32.2 (21.6, 42.8) ***	19.5 (10.5, 28.5) ***
Serious Mental Illness	164	37.6 (26.9, 48.3) **	20.5 (11.3, 29.7) **
No Mental Health Condition	4826	15.5 (14.2, 16.8)	6.6 (5.7, 7.5)

Unweighted frequencies (*n*) and weighted prevalence rates are reported. 95% confidence intervals are provided in parentheses. Asterisks indicate significant associations between MHC and ENDS use as determined by Rao-Scott χ^2^ tests, * *p* < 0.05; ** *p* ≤ 0.01; *** *p* ≤ 0.001. Serious Mental Illness is defined as reporting a diagnosis of at least one of the following mental health conditions: Bipolar Disorder, Schizoaffective Disorder, or Schizophrenia.

**Table 5:** Prevalence of ENDS use as a quit method by lifetime mental health condition and smoking status.

Mental Health Condition	Overall	Former Smoker (*n* = 1686)	Current Smokers (*n* = 1281)
Switched Completely to ENDS			
	**	**	
Mental Health Condition	14.3 ( 10.6, 17.9)	11.4 (6.6, 16.2)	17.9 (12.3, 23.4)
No Mental Health Condition	9.2 (7.7, 10.7)	5.3 (4.0, 6.6)	18.0 (14.3, 21.8)
Overall	10.3 (8.9, 11.8)	6.4 (5.0, 7.8)	18.0 (14.9, 21.1)
Substituted Some Regular Cigarettes with ENDS			
	**	**	
Mental Health Condition	23.1 (18.8, 27.4)	13.7 (8.2, 19.3)	35.0 (28.2, 41.7)
No Mental Health Condition	16.0 (14.0, 18.0)	7.3 (5.5, 9.2)	35.6 (31.0, 40.1)
Overall	17.6 (15.7, 19.4)	8.6 (6.7, 10.4)	35.4 (31.6, 39.2)

Weighted prevalence rates are reported, with 95% confidence intervals in parentheses. Asterisks indicate significant associations between MHC and ENDS use as a quit method among the overall sample and by smoking status, as determined by Rao-Scott χ^2^ tests, ** *p* ≤ 0.01.

**Table 6:** Susceptibility to ENDS use (among participants who have never used ENDS).

Mental Health Condition	Overall	Non-Smokers (*n* = 2485)	Former Smokers (*n* = 1234)	Current Smokers (*n* = 437)
Do you think you will try an electronic vapor product soon? (definitely/probably yes)				
	**		**	*
Mental Health Condition	4.4 (2.4, 6.5)	0.8 (0.0, 1.6)	1.8 (0.0, 3.6)	26.9 (14.2, 39.5)
No Mental Health Condition	2.0 (1.3, 2.8)	1.7 (0.8, 2.5)	0.3 (0.0, 0.6)	13.8 (8.1, 19.5)
Overall	2.4 (1.8, 3.1)	1.5 (0.8, 2.3) **^a^**	0.5 (0.1, 1.0) **^b^**	17.5 (11.9, 23.0) **^c^**
Have you ever been curious about using an electronic vapor product? (definitely/probably yes)				
	***		**	*
Mental Health Condition	14.5 (11.3, 17.7)	7.2 (4.3, 10.1)	13.7 (8.2, 19.2)	49.4 (36.0, 62.7)
No Mental Health Condition	7.8 (6.6, 9.0)	6.1 (4.7, 4.5)	5.9 (3.9, 7.9)	33.4 (25.8, 41.0)
Overall	8.9 (7.8, 10.0)	6.3 (5.0, 7.5) **^a^**	7.3 (5.4, 9.2) **^a^**	37.8 (31.1, 44.5) **^b^**
If one of your best friends were to offer you an electronic vapor product, would you try it? (definitely/probably yes)				
	***		***	
Mental Health Condition	15.0 (11.8, 18.2)	9.7 (6.2, 13.3)	9.4 (5.4, 13.4)	51.8 (38.5, 65.0)
No Mental Health Condition	8.2 (7.0, 9.4)	6.8 (5.3, 8.3)	3.3 (2.2, 4.5)	44.2 (36.3, 52.0)
Overall	9.3 (8.2, 10.5)	7.2 (5.8, 8.6) **^a^**	4.4 (3.2, 5.6) **^b^**	46.3 (39.6, 53.1) **^c^**

Percentages with non-matching letter superscripts are significantly different across smoking status, *p* < 0.05, as determined by Rao-Scott χ^2^ tests. Asterisks show significant associations between MHC and susceptibility to ENDS use among the overall sample and by smoking status, * *p* < 0.05; ** *p* ≤ 0.01; *** *p* ≤ 0.001. 95% confidence intervals are provided in parentheses.
